# Structural empowerment of midwifery students following simulation-based training of management of obstetric emergencies

**DOI:** 10.1186/s12909-023-04365-3

**Published:** 2023-05-23

**Authors:** Mojgan Janighorban, hojatollah yousefi, Nikoo yamani

**Affiliations:** 1 Nursing and Midwifery Care Research Center, Isfahan University of Medical Sciences, Isfahan, Iran; 2 Department of Adult Health Nursing, School of Nursing and Midwifery, Nursing and Midwifery Care Research Center, Isfahan University of Medical Sciences, Isfahan, Iran; 3 Department of Medical Education, Educational Development Center, Medical Education Research Center, Isfahan University of Medical Sciences, Isfahan, Iran

**Keywords:** Simulation, Structural empowerment, Midwifery, Midwifery students, Obstetric, Emergencies

## Abstract

**Background:**

Having the ability of managing obstetric emergencies is the necessary capability for providing care during labor and delivery.Simulation is considered to be a valuable strategy for empowering midwifery students in managing emergencies. So, this study was conducted to determine the structural empowerment of midwifery students following the simulation-based training of management of midwifery emergencies.

**Methods:**

This semi-experimental research was conducted from August 2017 to June 2019 in the Faculty of Nursing and Midwifery, Isfahan, Iran. 42 subjects of the third-year midwifery students were included in the study through convenience sampling method (n = 22 in the intervention group, n = 20 in the control group). Six simulation-based educational sessions were considered for the intervention group. Conditions for Learning Effectiveness Questionnaire, was used at the beginning of the study, one week after it and one year later. Repeated measures ANOVA was used to analyze the data.

**Results:**

In the intervention group, the significant difference was observed between the mean score of the students’ structural empowerment before and after the intervention (MD=-28.41, SD = 3.25) (p < 0.001), before and one year after the study (MD=-12.45, SD = 3.47) (p = 0.003), and immediately after and one year after the study (MD = 15.95,SD = 3.67) (p < 0.001). In the control group, no significant difference was observed. Before the intervention, there was no significant difference between the mean score of the students’ structural empowerment in the control and intervention groups (MD = 2.89, SD = 3.50) (p = 0.415); but, immediately after the intervention, the mean score of structural empowerment in the students of the intervention group was significantly higher than those of the control group (MD = 25.40, SD = 4.94 ) (p < 0.001). One year after the study, there was no significant difference between the two groups in terms of the mean score of the structural empowerment (MD = 6.89, SD = 3.58 ) (p = 0.061).

**Conclusion:**

Simulation contributed to the structural empowerment of midwifery students by providing the opportunity for personal and professional growth and strengthening of the formal and informal power in the area of midwifery emergency management but these benefits were not seen at one year.

## Background

According to the job description announced by the International Confederation of Midwives, midwives are responsible for providing the cares needed during pregnancy, delivery and after it. midwives are able to provide up to 87% of all the needs of mothers and newborns [1]. Having up-to-date knowledge of the potential complications of pregnancy and childbirth, and the ability of managing midwifery emergencies, including quick diagnosis of disorders and emergency situations, mastering the required skills, behaviors and capabilities, and timely implementation of critical and life-saving interventions are among the general competencies of a midwife and the necessary capabilities for providing care during labor and delivery [2].

Since complications and emergency situations are probable in 15% of deliveries, it is necessary to train skilled workers who are properly educated and confident in their skills to perform the related cares [3]. The existence of such competent and skilled personnel together with the provision of high-quality care can prevent the death of one million newborns and half of all maternal deaths each year [4].

However, based on the evidence and from the viewpoint of the experienced midwives, midwifery graduates lack some empowerments necessary for professional duties and responsibilities [5]. Midwifery students also believe that self-confidence and more education are the requirements of managing emergency and complicated situations [6].

The use of new and innovative technology and techniques such as film, simulation and mobile applications can strengthen the quality of midwifery education with regard to the management of midwifery emergency situations [7]. As an active educational strategy, simulation can provide students with the opportunity of learning, facing the challenges, and acquiring personal and professional skills in a safe and supportive environment. Simulation, thus, can be used to increase the knowledge, motivation and self-confidence of midwifery students, strengthen their thinking power, practical skills, decision-making, management and leadership and improve the safety of patients and the effectiveness of care [8].

Therefore, simulation is considered to be a valuable strategy for empowering midwifery students in managing midwifery emergencies. Structural empowerment refers to the ability of an organization in empowering individuals or groups in the workplace. According to Kanter’s theory of structural empowerment, power and opportunity are two structural features of an organization which affect the ability of individuals to access empowerment sources and are necessary for one’s success in their work. Power means access to support, information, resources and opportunity, that is, having the chance of learning and personal and professional growth. Access to these structures of empowerment is affected by the level of formal and informal power that a person has in an organization [9].

structural empowerment of students has a significant and positive correlation with their capabilities [10]. Therefore, according to the evidence, provision of structural empowerment experiences in the management of midwifery emergencies through using simulation method can be the basis for acquiring the professional skills that midwifery students need to manage complex and emergency midwifery situations. Therefore, this study was conducted to determine the structural empowerment of midwifery students following the simulation-based training of management of midwifery emergencies.

## Methods

**Fig. 1:**
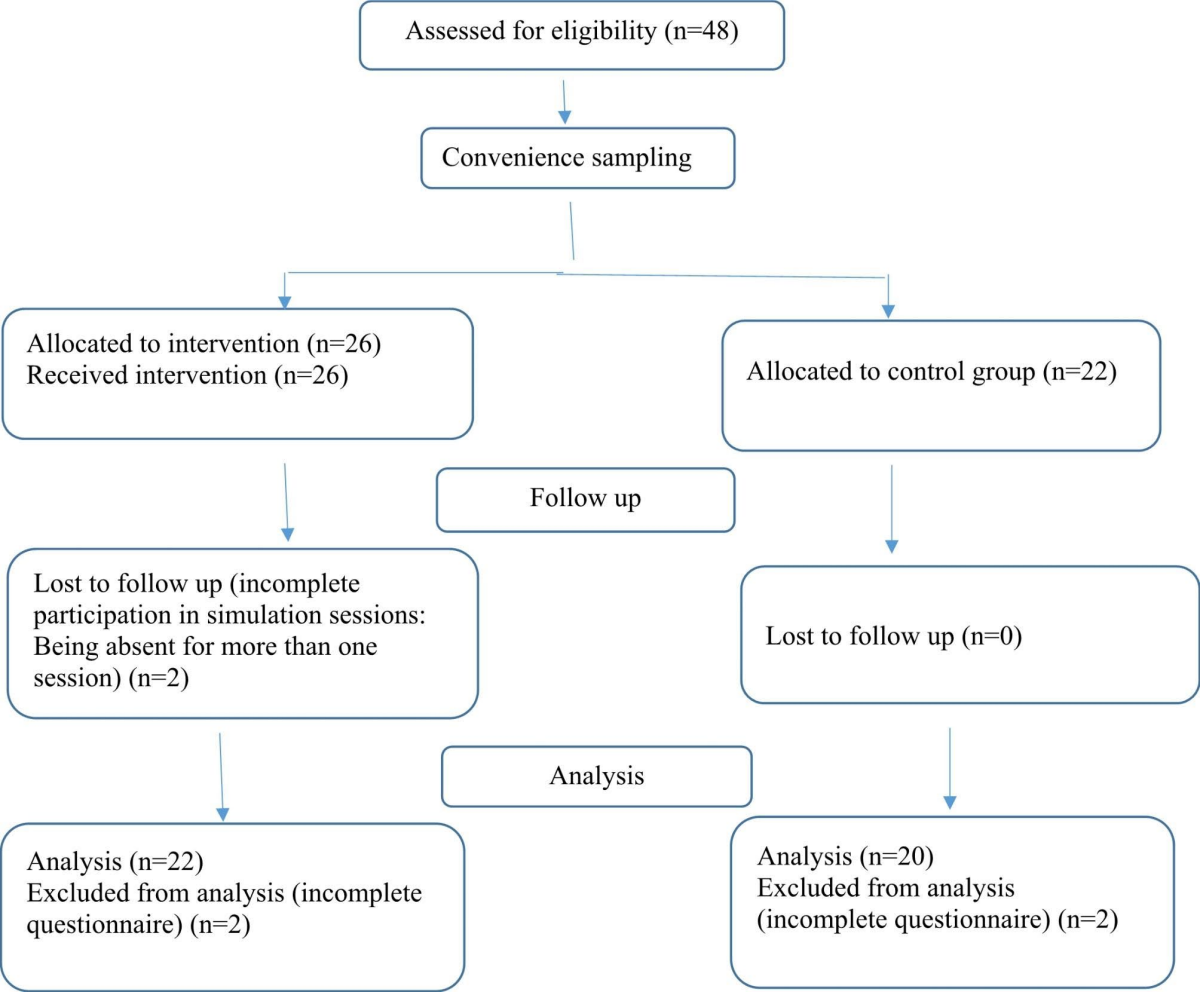
Flow diagram of study

This semi-experimental research was conducted from August 2017 to June 2019. The participants of the study were third-year undergraduate midwifery students in the Faculty of Nursing and Midwifery, Isfahan University of Medical Sciences, Iran. They were included in the study through convenience sampling method. All third-year midwifery students of this faculty in the summer of 2017 were invited as the intervention group (n = 26) and all the third-year students in the summer of 2018 as the control group (n = 22). Inclusion criteria were willingness to participate in the study, completion of the 6th semester of midwifery undergraduate course and passing all theory classes. Exclusion criteria included unwillingness to cooperate at any stage of the study, incomplete participation in simulation sessions, and incomplete questionnaires (Fig. 1).

### Implementation of the intervention

In this study, 6 simulation-based educational sessions were delivered for the intervention group, which were held in the clinical skills lab of the mentioned faculty. The sessions were held during a week for 5-6-person groups. The design of the simulation sessions, the relevant components and their educational content has been based on the collection of articles in “Standards of Best Practice: Simulation Standard 1–9”, which are the educational standards in simulated environments and sessions [11]. The specific educational content of the sessions was also extracted from the latest midwifery guidelines. The scenario of the simulation sessions was also written using the templates for compiling simulation scenarios in the area of medical simulation [12] (Wilson et al., Ross).

In this study, during 6 sessions, clinical management of the most important midwifery emergencies [13–15] was taught using high- and low-fidelity simulation, and clinical management of these situations was practiced by the students. The content of the sessions was as follows (Table 1):

**Table 1:** The content of simulation -based educational sessions

	Session’s title	Simulation fidelity	Intervention tools
1	Clinical management of episiotomy repair and wide ruptures of the vaginal floor	High-fidelity simulation	beef tongue
2	Clinical management of postpartum hemorrhage	High-fidelity simulation	Mannequin, artificial blood, leather gestational sac, jelly cotiledones and placenta membranes
3	Clinical management of umbilical cord prolapse	Low-fidelity simulation	Mannequin, doll, balloon, cellophane, socks
4	Clinical management of shoulder dystocia	Low-fidelity simulation	Mannequin
5	Clinical management of neonatal resuscitation	Low-fidelity simulation	Mannequin
6	Clinical management of eclampsia	Low-fidelity simulation	Mannequin

In each of these sessions, the students were taught the clinical management of the intended clinical situation step-by-step and based on the latest protocols and scientific texts. Then, the students were asked to practice and repeat the required skills individually and in groups. Next, the students demonstrated their skills in managing the intended clinical situation by participating in a scenario. In all of the sessions, the role playing of the students in the simulated situation was filmed by a camera so that after the simulated scenario, the students could review their function during a question and answer session. After completing the scenarios by the students, in the question and answer session, their function in clinical situations was analyzed by the researchers and the students themselves, and the necessary feedback was given to them. In order to apply high-fidelity simulation, beef tongue was used to teach and practice the repair of episiotomy and wide ruptures of the vaginal floor, and the students performed anesthesia in the perineum, episiotomy incision and its repair, and also repaired wide ruptures of the vaginal floor. In order to simulate the uterine massage after childbirth, the postpartum uterus moulage was used, in which artificial blood was injected and the uterus was massaged. Artificial blood was used for better simulation of the postpartum bleeding scene and made bleeding estimation possible. Artificial blood was made by mixing 36% glycerin and 64% water, which has the same density as natural blood. Ferric chloride and potassium thiocyanate were added to this solution and one liter of artificial blood was prepared for each session. In order to perform the courage, a leather bag that was prepared similar to the postpartum uterus, as well as paraffin jelly for the simulation of the cotiledones were used. With the help of these cotiledones, which were simulated with paraffin, the students could experience removing the cotyledon while doing the courage. Paraffin jelly was used to simulate placental membranes. Socks, balloons, cellophane plastic and baby dolls were used to practice amniotomy, which provided the opportunity of experiencing amniotomy and the care needed after it. In the present study, the control group participated in the regular educational programs of the faculty. Both groups completed the structural empowerment questionnaire at the beginning of the study, one week after it and one year later.

### Tools

Conditions for Learning Effectiveness Questionnaire, which evaluates the level of structural empowerment, was the tool for measuring structural empowerment in this research. This questionnaire is a modified form of the Conditions of Work Effectiveness scale, which was prepared by Laschinger et al. to be used in educational environments [16]. The tool includes the 6 sub-scales of support access (7 items), opportunity to learn and progress (6 items), information access (6 items), resource access (5 items), informal power (4 items) and formal power (2 items). Each item is scored using a 5-point Likert scale, and the range of scores is between 30 and 150. Higher scores represent stronger perceptions of working in an empowered work environment. Scores ranging from 30 to 65 are described as low levels of empowerment, 70 to 110 as moderate levels of empowerment, and 115 to 150 as high levels of empowerment. The validity and reliability of the structural empowerment questionnaire have been investigated and confirmed [17]. Cronbach’s alpha for this scale was equal to 0.81 in the present study.

### Data analysis

Statistical analysis was performed at two descriptive and inferential levels. Mean and standard deviation were used in the descriptive level. Repeated measures ANOVA (2 × 3) was used in the inferential level. Bonferroni’s post-hoc test was used to make pairwise comparisons. The basic assumptions of the model, such as normal error distribution and homogeneity of error variance, were examined and confirmed by the Shapiro-Wilk’s and Levene’s tests, respectively. The tests were performed at a 5% error level using SPSS software version 19.

### Ethical considerations

This research was approved by the Ethics Committee of Isfahan University of Medical Sciences with the ethics code of IR.MUI.RESEARCH.REC.1397.221. All methods were performed in accordance with the relevant guidelines and regulations. The participation of the participants in this study was completely voluntary and informed written consent was obtained from them. The questionnaires were anonymous and the participants were assured that their information would remain confidential.

## Results

In this study, two participants with incomplete participation in simulation sessions and four participants with incomplete questionnaire (n = 2 in the intervention group and n = 2 in the control group) were excluded and finally 42 midwifery students participated and were divided into two groups of control (n = 20) and intervention (n = 22). The subjects of the control group were between 21 and 24 years old with the mean age of 21.90 ± 0.79 years, and those of the intervention group were between 20 and 24 years old and the mean age of 21.50 ± 1.01 years. Based on the result of the Mann-Whitney test, no significant difference was observed in the age of the students of the two groups (p = 0.066, Z = 1.839). The grade point average of students in the control group and the intervention group was 16.08 ± 1.49 and 16.39 ± 0.99, respectively. According to the result of the independent t-test there was no significant difference between the grade point average of the students of the two groups (p = 0.429, t(40)= -0.800).

The mean score of structural empowerment obtained by the students of two groups and the results of repeated measures ANOVA are shown in Table 2.

**Table 2:** The mean score and standard deviation of the structural empowerment in the two groups during the pre-test and post-test stages and the results of the repeated measures ANOVA

		Mean ± sd			Repeated measures ANOVA		
Variable	Group	Before the study	one week after the study	One year after the study	Time	Group	Interaction effect
Structural empowerment	Intervention	92.64 ± 12.97	121.05 ± 18.03	105.09 ± 11.35	p < 0.001	p < 0.001	p < 0.001
	Control	89.75 ± 9.21	95.65 ± 13.36	98.20 ± 11.85	(η^2^ = 0.372)	(η2 = 0.298)	(η^2^ = 0.222)

Based on the results of repeated measures ANOVA, the measurement time effect (η2 = 0.372, p < 0.001, F (2,80) = 23.71), the intervention group effect (η2 = 0.298, p < 0.001, F (1.40) = 16.99) and time-by-group interaction effect (η2 = 0.222, p < 0.001, F (2,80) = 11.43) on the structural empowerment of the students were significant at the level of 5% error. The results of Bonferroni’s post-hoc test with regard to the interaction effect indicated that in the intervention group, the mean score of structural empowerment before the intervention was significantly lower than immediately after (p < 0.001) and one year after the intervention (p = 0.003). Moreover, the mean score of structural empowerment one year after the intervention was significantly lower than immediately after the intervention (p < 0.001). By contrast, regarding the control group, no significant difference was observed between the mean score of the students’ structural empowerment before and after the intervention (p = 0.274), before and one year after the intervention (p = 0.076), and immediately after and one year after the intervention (p = 1.00) (Table 3). Additionally, as the results of inter-group comparisons through using Bonferroni’s post-hoc test showed, before the intervention, there was no significant difference between the mean score of the students’ structural empowerment in the control and intervention groups (p = 0.415); but, immediately after the intervention, the mean score of structural empowerment in the students of the intervention group was significantly higher than those of the control group(p < 0.001). One year after the intervention, there was no significant difference between the two groups in terms of the mean score of the structural empowerment (p = 0.061) (Table 4).

**Table 3:** The results of Bonferroni’s post-hoc test comparing the mean scores of the students’ structural empowerment between the three stages of before, immediately after and one year after the intervention in the control and intervention groups

Group		Variable Time	Structural empowerment		
			Mean difference	Standard error	p-value
Intervention	Before-immediately after the intervention		-28.41	3.25	< 0.001
	Before-one year after		-12.45	3.47	0.003
	Immediately after-one year after		15.95	3.67	< 0.001
Control	Before-immediately after the study		-5.90	3.41	0.274
	Before-one year after		-8.45	3.64	0.076
	Immediately after-one year after		-2.25	3.85	1.000

**Table 4:** The results of Bonferroni’s post-hoc test comparing the mean scores of the students’ structural empowerment in the control and intervention groups before, immediately after and one year after the stydy

Variable Time	Structural empowerment		
	Mean difference	Standard error	p-value
Before the study	2.89	3.50	0.415
Immediately after the study	25.40	4.94	< 0.001
One year after the study	6.89	3.58	0.061

## Discussion

The present study was conducted to determine the structural empowerment of midwifery students following simulation-based training of management of obstetrics emergencies. As the results indicated, in the intervention group, immediately after and one year after the intervention, the mean score of structural empowerment was significantly higher than before the intervention. Before the intervention, both groups were at an average level of empowerment. However, the intervention was able to improve the empowerment of participants in the intervention group to a high level, which indicates the success of the intervention. In the control group no significant difference was observed in the level of their feeling of structural empowerment during the study. A review of the related literature showed that simulation had led to positive results in different target groups. Carolan-Olah et al. conducted a qualitative evaluation of midwifery students after participating in the simulation of newborn resuscitation. According to the results, knowledge, confidence and skills of these students in managing this emergency situation improved and these students unanimously believed that active participation in simulation sessions with longer periods of time would strengthen their knowledge and self-confidence [18]. Kato and Kataoka showed that postpartum hemorrhage management education with the help of simulation improved the knowledge and performance of midwives with 2 or 3 years of clinical work [19]. Participating in three simulation scenarios of managing shoulder dystocia, breech delivery and postpartum hemorrhage has improved the skills and capabilities of a multidisciplinary group of health professionals, leading to very satisfying experiences for them [20].

Opportunity is a fundamental structure in the achievement of empowerment. Therefore, the provision of opportunity for acquiring knowledge and clinical work skills, and gaining clinical experience is one of the factors which facilitate the achievement of competence in managing midwifery emergencies. However, according to midwives, the increase of students with different disciplines in clinical environments leads to competition between midwifery students and other students for gaining learning opportunities. Furthermore, differences of clinical environments in providing opportunities for learning and gaining experience, as well as different approaches to childbirth management in these environments (medicalized midwifery care versus normal or physiological birth) cause concern about reduced learning opportunities for midwifery students, especially in non-prevalent situations [21].

According to Changuiti et al., simulation has provided the opportunity for learning simple to complex clinical situations in a safe and semi-real environment for midwifery students [22]. In their systematic review, Yucel et al. indicated that simulation, as an educational tool, improved the implementation of technical skills in educational and clinical environments. Moreover, simulation led to better implementation of techniques and maneuvers for shoulder dystocia and postpartum bleeding in shorter periods of time that, consequently, improved neonatal outcomes [23]. From the viewpoint of students, gaining practical experience, achieving self-confidence, having equal learning opportunities for everyone, having enough time, the possibility of trial and error, and bridging theory to practice are the advantages of simulation-based educational programs in the field of midwifery [24]. Simulation also allows students to interact with classmates, peers and professors. In these calm environments, they learn communication and teamwork skills so that they can make good use of the previously acquired experiences in turbulent emergency situations. Providing the opportunity of focusing on communication skills and practicing delegated responsibilities in stressful situations, simulation leads to the evolution of professional roles for individuals [25].

Therefore, it seems that in our study, simulation contributed to the structural empowerment of individuals by providing the opportunity for personal and professional growth in the area of midwifery emergency management. Furthermore, gaining awareness of important issues in midwifery emergency management, having enough time to learn midwifery emergency management, sharing experiences and skills with classmates and professors during current interactions between individuals, obtaining information and resources and, finally, achieving structural empowerment were the opportunities facilitated by simulation. Active participation of students in simulation sessions and facing the related challenges, receiving feedback, useful reminders and being encouraged by the instructor, analyzing the performance of individuals in the simulated situation by the students themselves and the professor at the end of the sessions, which were filmed, support of the classmates, sharing efforts and learning beside them were other facilitating factors in our study which led to the achievement of structural empowerment. Since interaction with peers is the basis for gaining informal power and the acquisition of personal and professional power is the basis for achieving formal power, the implementation of the simulation program in our study also provided the opportunity for interacting with the instructors and classmates, and gaining skills of communication with others provided the basis for informal power. In this environment, the students experienced the support of the instructors and their classmates and, gaining competence in midwifery work, they were also provided with the opportunity of obtaining formal power. Accordingly, strengthening of the formal power and informal power, together with access to resources, support and information, provided the context for improving the structural empowerment of the subjects of the intervention group.

Based on the results of our study in the intervention group, the mean score of the students’ structural empowerment one year after the intervention was significantly lower than immediately after the intervention. One year after the intervention, the mean score of the structural empowerment of the intervention group was higher than that of the control group, but the difference was not statistically significant. In this regard, simulation courses in the field of management of shoulder dystocia and singleton vaginal breech delivery for obstetricians and midwives were held in Hong Kong. The results showed that although the knowledge and skills of participants increased immediately after and one year after the simulation, knowledge and skills decreased by the passage of time. In these studies, the knowledge and skills of the participants in managing clinical situation had decreased one year after the simulation that the results of our study are in line with these results [26, 27]. Therefore, repetition of educational courses based on the simulation of management of midwifery emergencies can revive the empowerment of students.

### Limitations

As our participants were a convenience sample of two consecutive years, this study may not be adequately powered to detect differences in the structural empowerment at one year.

## Conclusion

Through simulation-based education, midwifery students can be provided with learning opportunities, resources, information and support they need to manage midwifery emergencies. However, these educational courses should be repeated at regular intervals in order to maintain the acquired empowerment of the students in managing emergency midwifery situations.

## Data Availability

The datasets used and/or analysed during the current study available from the corresponding author on reasonable request.
